# Comparison of two low-fat diets, differing in protein and carbohydrate, on psychological wellbeing in adults with obesity and type 2 diabetes: a randomised clinical trial

**DOI:** 10.1186/s12937-018-0367-5

**Published:** 2018-06-15

**Authors:** Nerylee Ann Watson, Kathryn Ann Dyer, Jonathan David Buckley, Grant David Brinkworth, Alison Mary Coates, Gaynor Parfitt, Peter Ranald Charles Howe, Manny Noakes, Karen Joy Murphy

**Affiliations:** 1 Alliance for Research in Exercise, Nutrition and Activity, Sansom Institute for Health Research, University of South Australia, GPO Box 2471, Adelaide, SA 5001 Australia; 2 Commonwealth Scientific and Industrial Research Organization - Health and Biosecurity, PO Box 10041, Adelaide, SA 5000 Australia; 3 Clinical Nutrition Research Centre, School of Biomedical Sciences and Pharmacy, University of Newcastle, University Drive, Callaghan, NSW 2308 Australia; 4 Institute for Resilient Regions, University of Southern Queensland, Springfield, QLD 4300 Australia

**Keywords:** Type 2 diabetes, Psychological wellbeing, Quality of life, Emotional distress, Weight loss, Weight maintenance, Dietary protein

## Abstract

**Background:**

Although higher-protein diets (HP) can assist with weight loss and glycemic control, their effect on psychological wellbeing has not been established. The objective of this study was to compare the effects of a HP and a higher-carbohydrate diet (HC), combined with regular exercise, on psychological wellbeing both during weight loss (WL) and weight maintenance phases (WM).

**Methods:**

In a parallel RCT, 61 adults with T2D (mean ± SD: BMI 34.3 ± 5.1 kg/m^2^, aged 55 ± 8 years) consumed a HP diet (29% protein, 34% carbohydrate, 31% fat) or an isocaloric HC diet (21%:48%:24%), with moderate intensity exercise, for 12 weeks of WL and 12 weeks of WM. Secondary data evaluating psychological wellbeing was assessed using: Problems Areas in Diabetes (PAID); Diabetes-39 Quality of Life (D-39); Short Form Health Survey (SF-36); Perceived Stress Scale-10 (PSS-10) and the Leeds Sleep Evaluation Questionnaire (LSEQ) at Weeks 0, 12 and 24 and evaluated with mixed models analysis.

**Results:**

Independent of diet, improvements for PAID; D-39 diabetes control; D-39 severity of diabetes; SF-36 physical functioning and SF-36 general health were found following WL (*d* = 0.30 to 0.69, *P* ≤ 0.04 for all) which remained after 12 weeks of WM. SF-36 vitality improved more in the HP group (group x time interaction *P* = 0.03). Associations were seen between HbA1c and D-39 severity of diabetes rating (*r* = 0.30, *P* = 0.01) and SF-36 mental health (*r* = − 0.32, *P* = 0.003) and between weight loss and PAID (*r* = 0.30, P = 0.01).

**Conclusion:**

Several improvements in diabetes-related and general psychological wellbeing were seen similarly for both diets following weight loss and a reduction in HbA1c with most of these improvements remaining when weight loss was sustained for 12 weeks. A HP diet may provide additional increases in vitality.

**Trial registration:**

The trial was prospectively registered with the Australian New Zealand Clinical Trials Registry (ACTRN 12613000008729) on 4 January 2013.

## Background

By 2030, it is projected that 7.7% of the adult population globally will have diabetes with 90% being type 2 diabetes (T2D) [1]. T2D diagnosis instigates new life-long daily challenges with a heavy reliance on self-management. The *Diabetes Attitudes, Wishes and Needs second study* (DAWN2™) assessed psychosocial outcomes for 8596 adults with predominantly T2D (84%) across 17 countries where 45% of participants reported high levels of diabetes-related distress; 12% considered their quality of life (QoL) as ‘poor’ or ‘very poor’; and physical health (62%) and emotional wellbeing (46%) were considered to be adversely affected by their diabetes [2]. Furthermore, a cross-sectional analysis of adults with T2D reported 55% had poor quality of sleep and poor sleepers indicated significantly worse health related QoL (HRQoL) including emotional wellbeing and physical functioning [3]. Diabetes-related distress such as shock, guilt, anger, anxiety and helplessness is particularly high at diagnosis (85.2%), but psychological disturbances also continue long term [4], and individuals with T2D have a 2-fold greater risk of developing major depressive disorders [5]. In fact the fear of developing complications, the social and financial impact of managing their condition, and the burden placed on family members have been reported to underpin the majority of psychological problems [4]. In turn, these adverse psychological issues may lead to inadequate self-care, poor dietary intake, reduced physical activity, non-compliance with medications and less vigilance with blood glucose monitoring [6], thus contributing to poorer long-term health outcomes.

Diet and exercise form the basis of T2D self-management. An individual’s perception of their HRQoL is subjective; therefore improvements may result from achieving diabetes-specific management goals which have previously been difficult to reach (e.g. weight loss, better glycemic control, reduction in medications). Energy-restricted, low-fat diets with a higher protein-to-carbohydrate ratio have been shown to be effective for these management goals [7, 8]. However, there is a paucity of data examining the effects of HP diets on psychological wellbeing. A higher-protein intake of 0·8–1·2 g/kg body weight/day, even in energy-restriction, provides sustained satiety and maintains basal energy expenditure, in part, due to a high diet-induced thermogenesis and the preservation of fat-free-mass [9]. Additionally, compared to normal-protein diets, energy-restricted HP diets reduce hunger, the desire to eat and fast-food cravings in overweight women [10] and promote greater daily satiety and evening appetite control in overweight and obese men [11]. Therefore a HP diet may provide greater diet satisfaction and thus improving HRQoL through an enhanced sense of achievement and self-control.

To-date, most dietary intervention studies evaluating HRQoL are weight loss studies. However the benefits of weight loss for psychological wellbeing are not clear. Weight loss of at least 5 kg has been associated with HRQoL improvements in obese participants following a weight management intervention [12] and a meta-analysis of 117 weight loss treatments concluded that weight loss facilitated increases in self-esteem and this mediation was stronger with greater weight loss [13]. In contrast, results from the English Longitudinal Study of Ageing concluded that a significantly higher proportion of the weight loss group (lost ≥5% body weight) reported worsening psychological wellbeing than the groups who were weight stable or gained weight (regained ≥5% body weight) [14]. In a study of 117 overweight/obese men (BMI 31.2 kg/m^2^, age 49.6 years) without diabetes, similar improvements in mental and physical QoL health outcomes were reported after 52 weeks consuming either an energy-restricted higher-protein diet (HP: 31% protein; 36% carbohydrate) or an energy-matched higher-carbohydrate diet (HC: 21% protein; 48% carbohydrate) following weight loss (− 10.5% of body weight) [15]. However, whether similar responses are observed in T2D remains unclear.

Furthermore, a study examining the HRQoL effects of obese adults who regained weight (mean regain 10.1 ± 4.4%) following initial weight loss (mean loss − 18.8 ± 6.7%), showed HRQoL worsened as weight was regained in the same linear pattern seen for the HRQoL improvements produced during weight loss [16]. This suggests HRQoL changes in response to weight status, and benefits gained during weight loss may continue if weight loss is sustained, but there is a lack of evidence to support this hypothesis and, specifically, whether this occurs in individuals with T2D.

To expand the current literature, the objective of this study was to compare the effects of isocaloric HP and HC diets, combined with regular moderate intensity exercise, on psychological wellbeing and HRQoL outcomes in overweight and obese adults with T2D during a 12-week active weight loss phase followed by a 12-week weight maintenance phase where weight was stabilised.

## Methods

### Participants, study design and intervention

This paper reports a secondary analysis of data and the full protocol has been previously published [17]. In brief, 63 overweight and obese adults with T2D (BMI ≥ 25 kg/m^2^, aged 18–70 years, glycosylated haemoglobin; HbA1c 6.5–10.5%) were recruited from the general community for this 2-arm parallel study. Exclusion criteria included a diagnosis or treatment for any neurological or psychiatric condition except for stable antidepressant medication use (≥ 3 months). Participants were block-matched for age, sex and BMI by an independent investigator before being allocated to either a HP diet (*n* = 32) or an isocaloric HC diet (*n* = 31). The HP diet aimed for 32% of total energy as protein, 33% carbohydrate and 30% total fat (< 10% as saturated fat) and the HC diet aimed for 22% protein, 51% carbohydrate and 22% total fat (< 10% as saturated fat). The initial 12 weeks was a weight loss phase which was followed by a 12-week weight maintenance phase whilst preserving the prescribed macronutrient profiles. Participants were required to complete daily semi-quantitative food records throughout the intervention and individual consultations with a research dietitian were conducted every two-weeks throughout the study. Participants were prescribed to perform a minimum of 30 min of moderate intensity aerobic exercise of their choice for at least 5 days per week (150 min/week). Data collection was completed in January 2014.

### Clinical assessments

After an overnight fast, participants attended clinic assessment appointments at Week 0 (baseline), Week 12 (following weight loss) and Week 24 (following weight maintenance). Body mass (to the nearest 0.01 kg) and height (to the nearest 0.1 cm) were measured with participants’ barefoot and wearing minimal clothing. BMI was calculated using the formula: mass (kg)/height (m) ^2^. Venous blood samples were obtained for HbA1c and were analyzed by an accredited commercial pathology laboratory (SA Pathology).

### Questionnaires

Psychological wellbeing and quality of life questionnaires were completed during the breakfast break following clinical assessments and were assessed using five self-administered questionnaires: two diabetes-specific instruments and three generic instruments.

The Problems Areas in Diabetes (PAID) questionnaire measures diabetes-specific emotional distress including guilt, fear, anger, depressed mood and worry [18]. Total scores range between 0 and 100 and a higher score expresses higher diabetes-related distress.

The Diabetes-39 Quality of Life Questionnaire (D-39) assess quality of life in individuals with diabetes and covers five subscales of health: diabetes control, energy and mobility, anxiety and worry, social burden and sexual functioning [19]. Possible scores for subscales range between 0 and 100 with higher scores indicating poorer health. Two additional single item questions are included in the questionnaire to gauge each participant’s perception of their overall quality of life and the severity of their diabetes. Both of these items have a possible score of 0 to 7 with a higher score for the overall quality of life indicating higher QoL and a lower score for the severity of diabetes indicating less severity.

The Short Form-36 v2 Health Survey™ (SF-36) measures different aspects of physical health (physical functioning; role limitations due to physical health; bodily pain and general health) and emotional health (vitality; social functioning; role limitations due to emotional health and emotional health) plus an overall physical component summary score and an overall mental component summary [20]. Each subscale has a score between 0 and 100 with higher scores indicating better health status.

The Perceived Stress Scale-10 (PSS-10) measures a person’s perceived stress and coping ability and is a useful tool to examine the role of stress in regards to diseases and behaviour disorders [21]. Total scores range from 0 to 40 and a higher score indicates greater stress.

The Leeds Sleep Evaluation Questionnaire (LSEQ) measures aspects of sleep and early morning behaviour [22]. A shortened version was used in this study to evaluate quality of sleep (QOS) which has a possible score between 0 and 100 with a higher score indicating a lower sleep quality.

Recall periods are ‘*over the past 4 weeks*’ except for the SF-36 subscales of physical function and general health and the PAID questionnaire which are considered in the context of ‘*currently*’. All questionnaires are validated [23–27] and Cronbach’s alpha coefficients confirm very good internal consistency within this study’s cohort: SF-36 (*α* = 0.83); PSS-10 (*α* = 0.91); LSEQ (*ɑ* = 0.84); PAID (*α* = 0.95) and D-39 (*ɑ* = 0.96).

### Statistical analysis

Statistical analyses were performed using SPSS version 24.0 (SPSS Inc., Chicago, IL). Missing data were addressed depending on the questionnaire and the corresponding instructions. For the D-39 subscales, mean substitution was used in accordance with the D-39 scoring instructions [19]; the SF-36 subscales were scored by QualityMetric Incorporated and the LSEQ questionnaire QOS subscale was calculated as the mean of two responses. Subscales which could not be calculated using these methods were treated as missing data in the mixed-effects models.

The PAID and the PSS-10 questionnaires provide a total score which is the sum of the responses given. At baseline, four participants (HP *n* = 3, HC *n* = 1) had one missing answer from a total of 20 responses in the PAID questionnaire (5% of the questionnaire) and 2 participants (HC *n* = 2) had one missing answer from a total of 10 responses in the PSS-10 questionnaire (10% of the questionnaire). It has been reported that statistical analysis should not be biased if missing data is not greater than 10% [28]. For the PAID and PSS-10 questionnaires, since any incomplete answers prevented a total score from being determined for subsequent inclusion in the statistical analysis model, in the absence of any formal remedies for dealing with missing data for these questionnaires, missing data were replaced with the mean score for that participant. Sensitivity analysis conducted both with and without the mean substitution data provided similar results. Data were checked for normality and skewed data was normalised prior to analysis: PAID, D-39 subscales of diabetes control, anxiety and worry and social burden by square root transformation, the D-39 subscales of energy and mobility and sexual functioning by log transformation and SF-36 subscales of role limitations due to physical health and physical function by reflect square root transformation. Differences between completers and non-completers and dietary and exercise data were compared using independent student *t* tests for continuous variables and chi-square tests for categorical variables and are reported as means ± standard deviations (SD). The effects of the different interventions over time were assessed using an intention-to-treat analysis (including all participants who commenced the study) using mixed-effects models with an unstructured covariance matrix. Treatment was the between-subject factor and time was the repeated within-subject measurement. Where there was a significant main effect, *post-hoc* comparisons were performed with Bonferroni’s adjustments for multiple comparisons to determine differences between group means. The analysis was repeated with sex included as a factor but values for both sexes are only reported where a group by time by sex interaction was found. Statistical significance was set at *P* <  0.05 (two-tailed). Results from the mixed-effects models are presented as mean ± SEM.

Where an outcome showed a significant change over time, the effect size (ES) was calculated after imputing the means, standard deviations and the correlation coefficient between the two means [29]. Including the correlation coefficient corrects for dependence between the means in repeated measures to allow comparisons for between-subjects studies using Morris and DeShon’s equation #8 [30]. Effect sizes (*Cohen’s d*) were considered as small (< 0.20), moderate (0.5) and large (0.8) [31]. Within subjects correlation coefficient analysis was conducted to identify associations between psychological wellbeing outcomes and body mass and HbA1c over the course of the study using univariate analysis of covariance as described by Bland and Altman [32]. The strength of a correlation was considered small (< 0.29), moderate (0.30 to 0.49) or large (0.5 to 1.0) [31].

## Results

### Participants

**Fig. 1:**
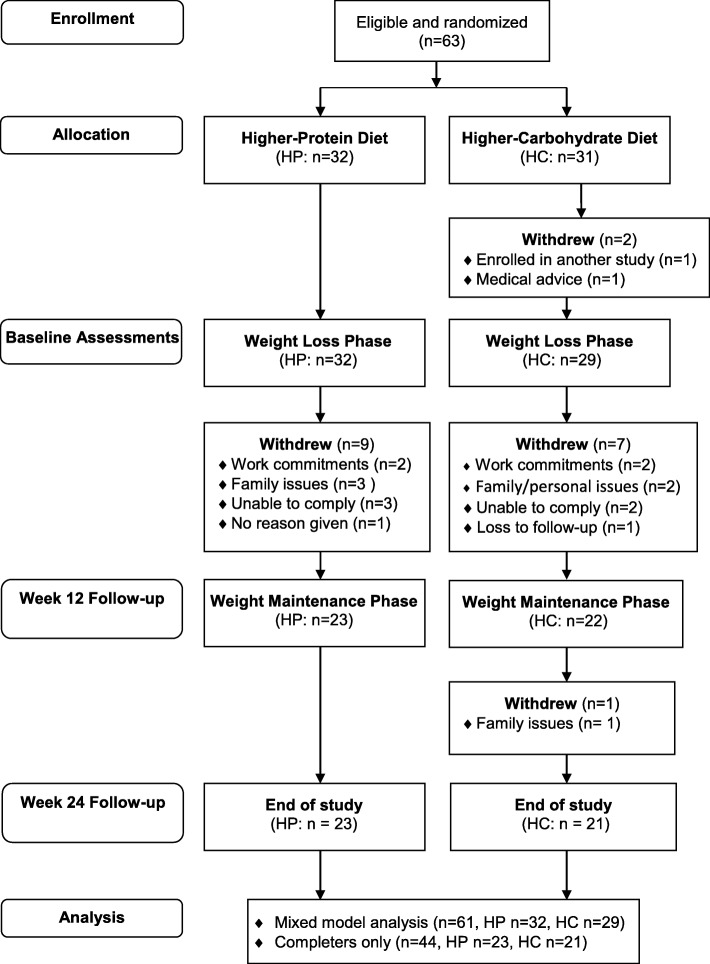
Study Flow Diagram

Participant characteristics, dietary and exercise data, body mass and HbA1c results have been reported in full elsewhere [8]. In brief, 61 participants commenced the study (HP: *n* = 32, HC: *n* = 29) whereby 17 withdrew (HP: *n* = 9, HC: *n* = 8) over the 24 weeks but were included in the mixed-model analysis (Fig. 1). All participants remained in their allocated diet group for analysis.

There were baseline differences between completers and non-completers for the SF-36 subscale of mental health (completers: 78.8 ± 14.6; non-completers: 68.8 ± 17.0, *P* = 0.03), the SF-36 physical component summary (completers: 50.3 ± 5.1; non-completers: 47.1 ± 5.1, *P* = 0.04) and LSEQ-QOS (completers: 38.68 ± 3.81; non-completers: 53.82 ± 6.10, P = 0.04). Therefore, in addition to the mixed model analyses which included data for all participants who commenced the study, sensitivity analysis was performed using data for completers only. For the SF-36 subscales of mental health and the physical component summary, both analyses showed the same pattern of outcomes and so the mixed-model analysis is reported. However there was a difference between the two methods for LSEQ QOS, therefore the mixed-model analysis could not be relied upon so only the completers analysis is reported. Baseline characteristics are shown in Table 1.

**Table 1:** Baseline sociodemographic and clinical characteristics of commencing participants by diet allocation

	HP diet (*n* = 32)		HC diet (*n* = 29)	
	*n*	(%)	*n*	(%)
Sex *n*, (%)				
Males	17	(53)	16	(55)
Females	15	(47)	13	(45)
	Mean	SD	Mean	SD
Age (years)				
Males	53	7	54	9
Females	56	10	57	6
Duration of T2D (years)	7.9	6.0	6.5	4.2
Body Mass (kg)	97.3	17.1	101.5	16.6
BMI (kg/m^2^)	34.3	5.4	34.4	4.7
HbA1c (%)	8.0	1.3	8.1	1.5
Problem Areas in Diabetes (PAID)				
PAID total score	23.9	19.7	25.6	18.5
Diabetes-39 Quality of Life Questionnaire				
Diabetes Control	24.1	18.0	26.9	18.9
Anxiety and Worry	36.2	21.9	34.7	18.9
Energy and Mobility	24.2	13.6	25.9	15.2
Social Burden	15.4	15.1	13.1	10.1
Sexual Functioning	21.4	26.7	24.5	26.4
Overall Quality of Life	4.7	1.3	4.9	1.3
Severity of Diabetes	3.5	1.7	3.7	1.5
SF-36 Health Survey				
Physical Functioning	80.5	16.1	80.7	14.1
Role Limitations - Physical	82.8	18.7	86.2	14.9
Bodily Pain	69.9	21.3	68.3	19.7
General Health	60.0	20.2	60.7	19.4
Vitality	56.1	17.6	58.0	18.4
Social functioning	87.5	17.4	86.2	19.9
Role Limitations - Emotional	87.4	14.1	85.7	18.0
Mental health	76.6	14.9	75.2	17.1
Physical Component Summary	49.1	5.7	49.6	4.9
Mental Component Summary	51.6	7.4	51.0	9.5
Perceived Stress Scale − 10 (PSS-10)				
PSS-10 total score	14.6	6.7	13.2	7.4
Leeds Sleep Evaluation Questionnaire				
Quality of sleep^†^	43.7	26.6	42.3	23.6

Data presented as numbers (*n*) and percentages (%) or mean values with standard deviations (SD). ^**†**^ HP: *n* = 20, HC: *n* = 20.

*Abbreviations: HP diet* Higher-protein diet, *HC* Higher-carbohydrate diet, *T2D* Type 2 diabetes, *BMI* Body mass index, *HbA1c* Glycosylated haemoglobin, *PAID* Problems Areas in Diabetes, *PSS-10* Perceived Stress Scale-10, *SF-36* Short Form-36 v2 Health Survey™

Food records indicated that, compared to the HC group, the HP group consumed more protein (mean ± SD of both phases: HP 28.7 ± 2.0, HC 20.5 ± 1.4%en, *P* <  0.001) and less carbohydrate (HP 34.0 ± 3.0, HC 48.1 ± 3.7%en, P <  0.001). Both groups achieved the exercise prescription, with no significant difference in the number of minutes of exercise performed per week between the groups (mean ± SD of both phases: HP 202 ± 89; HC 259 ± 141 min/week, *P* = 0.12). During the energy-restriction phase, weight loss was similar for both groups (HP: − 7.76 ± 0.72 kg; HC: − 7.61 ± 0.96 kg, *P* = 0.90) and remained stable during the weight maintenance phase (HP: − 0.94 ± 0.59 kg; HC: − 0.16 ± 0.61 kg, *P* = 0.36). During the weight loss phase, HbA1c decreased similarly in both groups (HP: − 1.53 ± 0.20%; HC: -1.30 ± 0.20%, *P* = 0.42) and remained stable during the weight maintenance phase (HP: 0.19 ± 0.16%; HC: -0.20 ± 0.16%, *P* = 0.65).

### Questionnaires

Results from the primary mixed-model analyses are shown in Table 2 (diabetes-specific questionnaires) and Table 3 (generic questionnaires). There was a group x time interaction for the SF-36 subscale vitality (*P* = 0.03). *Post-hoc* comparisons showed that vitality scores improved with the HP diet following weight loss (mean change: 12.33 ± 2.77, *P* <  0.001, *d* = 0.91) and remained higher than baseline at Week 24 (7.01 ± 2.57, P = 0.03, *d* = 0.47), whereas the HC group scores did not change significantly from baseline to Week 24 (5.74 ± 2.68, *P* = 0.11, *d* = 0.49). No other outcomes demonstrated a statistically significant difference between the diet groups over time (group x time interaction *P* ≥ 0·08) and there were no group effects for any outcomes (*P* ≥ 0.34).

**Table 2:** Diabetes-specific psychological wellbeing questionnaire scores at each time point (weeks 0, 12, 24) by allocated diet

				Weight Loss	Weight Maintenance	Complete Study	*P* value		
	Week 0	Week 12	Week 24	Mean Change (Weeks 0–12)	Mean Change (Weeks 12–24)	Mean Change (Weeks 0–24)	Group	Time	Group x Time
Problem Areas In Diabetes				−8.38 ± 1.75^a^	−2.63 ± 1.11	−11.01 ± 1.85^a^	0.84	< 0.001	0.08
HP	23.88 ± 3.38	18.03 ± 2.83	13.00 ± 2.39						
HC	25.56 ± 3.55	14.65 ± 2.93	14.42 ± 2.49						
Diabetes Quality of Life - 39									
Diabetes Control				−6.26 ± 2.19^b^	0.16 ± 1.56	−6.10 ± 1.82^b^	0.92	0.01	0.26
HP	24.20 ± 3.26	20.95 ± 2.29	19.66 ± 2.61						
HC	26.56 ± 3.43	17.29 ± 2.38	18.90 ± 2.72						
Anxiety and Worry				−4.83 ± 2.14	1.97 ± 2.07	−2.86 ± 2.18	0.55	0.14	0.65
HP	36.78 ± 3.66	32.94 ± 3.35	33.91 ± 3.55						
HC	34.28 ± 3.84	28.47 ± 3.50	31.43 ± 3.71						
Social Burden				−2.25 ± 1.42	0.78 ± 1.61	−1.47 ± 1.89	0.72	0.36	0.20
HP	15.38 ± 2.30	13.70 ± 1.96	11.53 ± 2.12						
HC	13.21 ± 2.42	10.38 ± 2.02	14.11 ± 2.21						
Energy and Mobility				−6.20 ± 1.52^a^	3.97 ± 1.63	−2.23 ± 1.26	0.92	< 0.001	0.52
HP	24.27 ± 2.53	19.63 ± 2.29	22.04 ± 2.49						
HC	25.52 ± 2.66	17.76 ± 2.40	23.28 ± 2.61						
Sexual Functioning^c^				−5.18 ± 2.55	1.45 ± 2.46	−3.73 ± 2.24	0.72	0.13	0.68
HP	22.42 ± 4.74	15.03 ± 3.95	19.01 ± 4.53						
HC	24.17 ± 4.88	21.21 ± 4.06	20.12 ± 4.70						
Overall Quality of Life^d^ (scores 0–7)				0.54 ± 0.19^b^	−0.21 ± 0.19	0.33 ± 0.21	0.84	0.03	0.36
HP	4.67 ± 0.23	5.46 ± 0.22	5.02 ± 0.23						
HC	4.89 ± 0.24	5.19 ± 0.23	5.20 ± 0.24						
Severity of Diabetes (scores 0–7)				−0.79 ± 0.19^b^	0.21 ± 0.19	− 0.58 ± 0.15^b^	0.88	< 0.001	0.15
HP	3.41 ± 0.29	3.01 ± 0.24	2.95 ± 0.28						
HC	3.69 ± 0.31	2.52 ± 0.25	3.01 ± 0.29						

Data reported as means ± SEM analyzed using mixed-models with group and time as fixed-factors. ^a^Significant difference < 0.001 level (two-tailed). ^b^Significant difference < 0.05 level (two-tailed). Post-hoc comparisons were performed with Bonferroni’s adjustments for multiple comparisons where a significant difference was seen. Unless indicated otherwise, scores range from 0 – 100. Lower scores signify an improvement in psychological wellbeing except ^d^ where higher scores indicate better QoL. Abbreviations: HP: higher-protein diet (*n* = 32); HC: higher-carbohydrate diet (*n* = 29) except for c (HP: *n* = 31; HC: *n* = 29)

**Table 3:** Generic psychological wellbeing questionnaire scores at each time point (weeks 0, 12, 24) by allocated diet

				Weight Loss	Weight Maintenance	Complete Study	*P* values		
	Week 0	Week 12	Week 24	Mean Change (Weeks 0–12)	Mean Change (Weeks 12–24)	Mean Change (Weeks 0–24)	Group	Time	Group x Time
SF-36 Health Survey^a^									
Physical Functioning^b^				3.23 ± 1.38^c^	0.14 ± 1.47	3.37 ± 1.52^c^	0.87	0.002	0.83
HP	80.44 ± 2.68	82.54 ± 3.17	84.73 ± 3.04						
M	87.89 ± 3.24	93.72 ± 3.86	92.89 ± 4.03	5.83 ± 2.60^c^	−0.83 ± 2.74	5.00 ± 2.82			
F	72.00 ± 3.42	70.24 ± 4.07	75.20 ± 4.25	−1.77 ± 2.64	4.96 ± 2.86	3.20 ± 2.87			
HC	72.00 ± 3.42	85.10 ± 3.30	83.18 ± 3.18						
M	86.94 ± 3.31	89.81 ± 3.81	85.52 ± 3.96	2.87 ± 2.34	−4.29 ± 2.54	−1.42 ± 2.55			
F	73.08 ± 3.67	80.01 ± 4.54	82.25 ± 4.89	6.93 ± 3.09	2.24 ± 3.54	9.17 ± 3.55^c^			
Role Limitations due to Physical Health				4.28 ± 1.81^a^	−5.15 ± 2.05	− 0.87 ± 1.99	0.88	0.03	0.31
HP	82.64 ± 3.01	89.08 ± 3.23	84.35 ± 3.75						
HC	86.21 ± 3.14	88.34 ± 3.34	82.77 ± 3.92						
Bodily Pain^b^				0.72 ± 3.79	−5.97 ± 3.57	−5.26 ± 2.35	0.83	0.05	0.88
HP	69.43 ± 3.66	68.55 ± 4.76	63.13 ± 4.43						
M	68.16 ± 5.00	78.83 ± 6.26	68.58 ± 6.12	10.67 ± 6.89	−10.25 ± 6.60	0.42 ± 4.26			
F	70.53 ± 5.27	57.26 ± 6.54	56.94 ± 6.46	−13.27 ± 7.17	−0.33 ± 6.92	−13.60 ± 4.32^c^			
HC	68.75 ± 3.84	71.05 ± 4.97	64.53 ± 4.64						
M	74.06 ± 5.10	76.14 ± 5.84	66.05 ± 6.02	2.08 ± 6.50	−10.09 ± 6.24	−8.01 ± 3.83			
F	61.59 ± 5.75	61.80 ± 8.06	65.67 ± 7.48	0.21 ± 8.73	3.87 ± 8.64	4.08 ± 5.65			
General Health				9.29 ± 2.49^c^	−1.19 ± 2.27	8.11 ± 2.33^c^	0.99	0.001	0.89
HP	59.77 ± 3.52	70.14 ± 3.23	68.05 ± 3.50						
HC	60.66 ± 3.67	68.88 ± 3.32	68.60 ± 3.65						
Vitality							0.76	0.001	0.03^d^
HP	56.09 ± 3.20	68.42 ± 2.69	63.10 ± 3.43	12.3 ± 2.8^d^	−5.3 ± 2.6	7.0 ± 2.6^c^			
HC	58.71 ± 3.36	60.83 ± 2.81	64.45 ± 3.59	2.1 ± 2.9	3.6 ± 2.8	5.7 ± 2.7			
Social Functioning				−3.16 ± 3.22	0.62 ± 3.55	−2.54 ± 2.04	0.69	0.34	0.76
HP	87.90 ± 3.31	86.06 ± 4.68	84.22 ± 3.78						
HC	86.21 ± 3.45	81.73 ± 4.81	84.80 ± 3.96						
Role Limitations due to Emotional Problems				0.61 ± 2.29	−2.93 ± 2.02	− 2.31 ± 1.90	0.68	0.27	0.96
HP	87.65 ± 2.84	88.77 ± 3.72	85.26 ± 3.78						
HC	86.08 ± 2.99	86.19 ± 3.88	83.85 ± 3.95						
Mental Health				2.59 ± 1.85	−1.65 ± 1.94	0.94 ± 1.75	0.54	0.39	0.68
HP	76.59 ± 2.83	80.32 ± 2.95	76.97 ± 2.89						
HC	74.77 ± 2.98	76.22 ± 3.09	76.27 ± 3.02						
Physical Component Summary^b^				1.82 ± 0.83	−0.93 ± 0.85	0.90 ± 0.75	0.67	0.10	0.79
HP	49.04 ± 0.95	50.69 ± 1.33	50.32 ± 1.27						
M	50.25 ± 1.25	54.36 ± 1.73	52.51 ± 1.67	4.10 ± 1.51^c^	−1.85 ± 1.57	2.26 ± 1.28			
F	47.61 ± 1.32	46.67 ± 1.81	47.72 ± 1.76	− 0.93 ± 1.55	1.05 ± 1.64	0.12 ± 1.30			
HC	49.80 ± 1.00	51.79 ± 1.39	50.31 ± 1.33						
M	51.51 ± 1.27	52.41 ± 1.65	49.52 ± 1.63	0.89 ± 1.38	−2.89 ± 1.45	− 1.99 ± 1.15			
F	47.44 ± 1.44	51.46 ± 2.17	52.98 ± 2.07	4.02 ± 1.96	1.52 ± 2.10	5.54 ± 1.68^c^			
Mental Component Summary				0.65 ± 1.13	− 0.55 ± 1.04	0.10 ± 0.86	0.49	0.83	0.29
HP	51.80 ± 1.49	53.68 ± 1.54	51.48 ± 1.53						
HC	51.03 ± 1.57	50.46 ± 1.60	51.56 ± 1.60						
Perceived Stress Scale − 10 (scores 0–40)^b^				−1.63 ± 0.73	1.65 ± 0.76	0.02 ± 0.99	0.38	0.02	0.85
HP	14.56 ± 1.24	12.80 ± 1.14	14.87 ± 1.50						
M	12.65 ± 1.69	11.88 ± 1.53	14.24 ± 2.00	−0.76 ± 1.35	2.36 ± 1.44	1.60 ± 1.80			
F	16.73 ± 1.80	13.85 ± 1.61	15.61 ± 2.10	−2.88 ± 1.41	1.76 ± 1.51	− 1.12 ± 1.88			
HC	13.24 ± 1.30	11.74 ± 1.19	12.98 ± 1.57						
M	13.13 ± 1.75	10.04 ± 1.49	10.44 ± 1.92	−3.09 ± 1.29	0.40 ± 1.34	− 2.69 ± 1.69			
F	13.39 ± 1.94	14.80 ± 1.88	17.65 ± 2.49	1.42 ± 1.68	2.85 ± 1.95	4.26 ± 2.28			
Leeds Sleep Evaluation Questionnaire									
Quality of Sleep^e^				−5.6 ± 2.6	1.7 ± 2.6	−3.9 ± 2.9	0.51	0.10	0.41
HP	39.3 ± 5.3	30.3 ± 4.5	32.9 ± 3.8						
HC	39.2 ± 5.4	37.0 ± 4.7	37.8 ± 4.0						

Data reported as means ± SEM analyzed using mixed-models with group and time as fixed-factors. ^d^Values for males (M) and females (F) are reported only where a significant group x time x sex interaction was found: SF-36 physical functioning (*P* = 0.04); SF-36 bodily pain (*P* = 0.02); SF-36 physical component summary (*P* = 0.002); PSS-10 (P = 0.03). ^a^Significant difference < 0.001 level (two-tailed). ^b^Significant difference < 0.05 level (two-tailed). Post-hoc comparisons were performed with Bonferroni’s adjustments for multiple comparisons where a significant effect was seen. Unless indicated otherwise, scores range from 0 – 100. Lower scores indicate an improvement in wellbeing except for ^c^ where higher scores indicate better QoL. ^e^Analysis on completers only (HP: *n* = 20, HC: *n* = 20)

Independent of diet group, several outcomes improved following 12 weeks of weight loss: PAID (*d* = 0.69, *P* < 0.001); D-39 subscales of diabetes control (*d* = 0.40, *P* = 0.04), energy and mobility (*d* = 0.58, *P* < 0.001), overall QoL (*d* = 0.37, *P* = 0.02) and severity of diabetes (*d* = 0.54, *P* = 0.001) and SF-36 subscales of physical functioning (*d* = 0.30, *P* = 0.01), role limitations due to physical health (*d* = 0.33, P = 0.03) and general health (*d* = 0.51, P = 0.001). At the end of the study (Week 24), after 12 weeks of weight maintenance, improvements from baseline remained significant only for PAID (*d* = 0.87, P < 0.001); D-39 subscales of diabetes control (*d* = 0.49, *P* = 0.01) and severity of diabetes (*d* = 0.53, *P* = 0.002) and SF-36 subscales of physical functioning (*d* = 0.29, P = 0.01) and general health (*d* = 0.49, *P* = 0.003).

When sex was added as a factor in the analysis, group by time by sex interactions were seen within two of the physical aspects of the SF-36, physical functioning (P = 0.04) and bodily pain (*P* = 0.02) and the overall the physical component summary (P = 0.002, Table 3). For physical functioning, males in the HP group reported a significant improvement (increase in scores) following the weight loss phase (*P* = 0.01) and the females in the HC group reported a significant improvement over the course of the study (*P* = 0.02). Changes in scores did not reach significance for the females in the HP group or the males in the HC group (*P* ≥ 0.29). For bodily pain, decreasing changes over time for the females in the HP group revealed significant worsening bodily pain from baseline to the end of the study (*P* = 0.01) whereas changes across time for males in both the HP and HC groups and females in the HC group did not reach significance (*P* ≥ 0.13). For the physical component summary, an increase in scores for the males in the HP group showed an improvement only following weight loss (*P* = 0.03) whereas the females in the HC group showed progressive improvement across all time points which was significant over the course of the study (*P* = 0.01). Scores for the females in the HP group and males in the HC group did not significantly change (*P* ≥ 0.16).

Although there was a time effect for the PSS-10 (*P* = 0.02) *post-hoc* comparisons did not show any significant changes over time points (*P* ≥ 0.09). There was a group x time x sex interaction for the PSS-10 (P = 0.03) but *post-hoc* comparisons did not show any significant changes over time for either sex in each group (*P* ≥ 0.06).

### Within-subject correlations

Over the 24-week study (Weeks 0–24) positive associations were seen between HbA1c and PAID (*r* = 0.26, P = 0.02), D-39 subscales of diabetes control (*r* = 0.23, P = 0.03), anxiety and worry (*r* = 0.26, P = 0.01), severity of diabetes (*r* = 0.30, P = 0.01) and PSS-10 (*r* = 0.23, P = 0.03). HbA1c was negatively associated with SF-36 subscales general health (*r* = − 0.26, P = 0.02), mental health (*r* = − 0.32, *P* = 0.003) and MCS (*r* = − 0.28, P = 0.01) when controlled for weight. Positive associations were seen between body mass and PAID (*r* = 0.30, P = 0.01) and LSEQ-QOS (*r* = 0.28, P = 0.01).

## Discussion

In this randomized controlled trial of overweight and obese adults with T2D, similar improvements occurred in general physical aspects of health as well as diabetes-specific emotional distress and QoL after consuming either a hypocaloric HP diet or HC diet combined with exercise training. Furthermore, when weight loss was stabilised and exercise maintained, many of the benefits were sustained. The initial 12 weeks of this study was a weight loss phase (mean loss - 7.8%). Given the lack of studies evaluating the effects of HP and HC diets in weight maintenance, this study was designed to reassess outcomes after a 12-week weight maintenance phase without the influence of further active weight loss. During this time period, weight and HbA1c were successfully stabilised for both groups and allocated macronutrient composition and physical activity were maintained.

The SF-36 emotional health subscale vitality was the only outcome to show a different response between the groups. The HP group exhibited a 22% improvement, equating to a very large magnitude of change (*d* = 0.91), compared to the HC group (4%) at the end of the weight loss phase (i.e. Week 12). Assessing the clinical importance of findings is subjective but an effect size of ≥0.5 has been suggested as clinically relevant for interpreting HRQoL results in chronic diseases [33], therefore this finding of improved vitality would appear to be clinically relevant. The reason for the greater improvement following the HP diet is unclear, but there are two possible explanations relating to the physiological effects of dietary protein. A 10-week weight loss study of overweight and obese women (45–56 years) compared low-fat diets (< 30% total fat) that were either HP (30% protein: 41% carbohydrate) or HC (16% protein: 58% carbohydrate) and found that the HP diet promoted greater satiety, higher perceived energy levels and fewer variations in blood glucose levels (BGL) than the HC diet [34]. Furthermore, fatigue in T2D has been associated with fluctuations in BGL, including acute hyperglycemia, which is not necessarily detected by HbA1c levels [35]. Higher carbohydrate loads can lead to a higher post-prandial BGL, therefore it is plausible the lower carbohydrate intake associated with the higher dietary pattern may have reduced fluctuations in BGL resulting in less tiredness and an increase in perceived vitality. However, this disparity between the diet groups was not sustained through the weight maintenance period, where HbA1c stabilised, with both groups achieving similar improvements at the end of the study (HP: *d* = 0.48, HC: *d* = 0.49). Although the overall change in HC was smaller than in HP and did not reach statistical significance, this may be due to a lack of statistical power. Nevertheless, the findings of this study in relation to effects on vitality suggest that a HP diet may assist in promoting vitality initially during weight loss while glycemic control is improving, but whether those benefits are maintained during weight maintenance are not conclusive.

A previous study reported improvements in PAID and all D-39 QoL subscales (except social burden) after 16 weeks on an energy-restricted diet (6-7 MJ/day) both with and without resistance training (3 times/week) in overweight and obese adults with T2D [36]. This is consistent with this current study which found both groups achieved similar improvements in diabetes-specific emotional distress (as indicated by reductions in PAID scores) and diabetes-related QoL (D-39) subscales of diabetes control, energy and mobility, overall QoL and severity of diabetes following weight loss. The magnitude of change following weight loss for PAID (*d* = 0.69); energy and mobility (*d* = 0.58) and severity of diabetes (*d* = 0.54) is noteworthy and of clinical relevance. These findings are not surprising as weight and glycemic control was substantially improved during this period and realizing these fundamental goals in diabetes management is a substantial achievement. At the end of the study, after weight maintenance, improvements remained significant for PAID (*d* = 0.87), D-39 diabetes control (*d* = 0.49) and severity of diabetes (*d* = 0.53) with effect sizes either larger or unchanged.

Independent of diet, the SF-36 subscales pertaining to the physical aspects of health showed significant improvement: physical functioning, role limitations due to physical health and general health. The effect was greatest for general health (*d* = 0.51, large). This is generally consistent with the Look AHEAD study which found significant improvements in physical health (only reporting SF-36 PCS) following an intensive lifestyle intervention (weight loss: − 9.0 kg; physical activity: ≥ 175 min/week) compared to a diabetes support and education program (without attention on diet and exercise, weight loss: − 0.9 kg) [37]. Following the weight maintenance phase, improvements from baseline remained significant for the SF-36 physical functioning and general health subscales with the overall effect size for general health remaining clinically relevant (*d* = 0.49), and similar to that found in this study.

Interestingly, sex interactions were seen for some of the physical elements of the SF-36 outcomes. Compared to males in the HC group, physical functioning and the overall physical component summary improved from baseline for males in the HP group although these changes were only significant following weight loss. Bodily pain did not significantly changes across time for males in either group. In contrast, a 52-week weight loss study conducted in adult men with T2D showed physical functioning improved similarly with both a HP and HC diet over all time points and bodily pain improved after 12 weeks and was sustained for a further 40 weeks [15]. Conversely, in the present study, for females, the HC group reported significant improvements for physical functioning and the overall physical component summary over the course of the study and the HP group reported worsening bodily pain. Although there is a lack of studies evaluating HP diets and these outcomes in females with T2D, in a previous study, non-significant changes in physical functioning and bodily pain scores were seen after both an energy-restricted HP diet (1.2–1.4 g/kg/day) and an energy-restricted HC diet (60–65% carbohydrate, 0.8-1 g/kg/day protein) in females with sarcopenia obesity (generalized loss of skeletal muscle mass and strength) [38]. It is difficult to explain these differential responses to the 2 dietary patterns tested between the genders in this current study but the results suggest a sex disparity and larger studies are needed to investigate this more comprehensively.

Although a small positive relationship was observed between weight and QOS, QOS values did not change significantly over the course of the study for either group. This is consistent with a previous cross-over study of adults (without T2D) who were overweight/obese, where QOS was not significantly different between the lower-protein (10% of energy: 0.5 g/kg/day); normal-protein (20% of energy: 1.0 g/kg/day) or higher-protein diets (30% of energy:1.4 g/kg/day) even though weight loss was similar [39]. However, results from the present study are in contrast to a parallel study by the same authors who reported quality of sleep improved on a HP diet (1.5 g/kg/day) compared to the normal protein diet (0.8 g/kg/day) despite weight loss being similar [39]. There are several possibilities for the differing results: (1) while the protein content for their normal protein diet was similar to our HC diet (mean for weeks 0–24: 0.8 g/kg/day), the protein content of their HP diets was higher than for our HP diet (mean for weeks 0–24: 1.3 g/kg/day) and there may be a threshold effect which requires an intake greater than 1.3 g/kg/day; (2) the protein source used to increase protein content in the other study was a milk protein concentrate which, as the authors noted, may have increased tryptophan levels, a precursor for serotonin synthesis and (3) the inclusion of those taking medications for sleep, although monitored, may have influenced the results whereas only one participant in our study took medication to aid sleep. A meta-analysis of 10 cross-sectional studies determined that poor QOS was associated with higher HbA1c levels and having a good QOS would reduce HbA1c by 0.35% [40]. In the present study we did not find an association between changes in HbA1c and QOS.

Within subjects correlation analysis showed a medium strength relationship whereby perception of the severity of diabetes and mental health improved with reductions in HbA1c, whereas weight loss was moderately associated only with a reduction in diabetes-related emotional distress. Without a suitable control group for comparison, it is only possible to speculate that achieving better glycemic control induces a greater influence on psychological wellbeing and QoL in T2D than weight loss. This may be, in part, from understanding that tighter glycemic control minimizes diabetes-related complications. Future research with larger and longer studies could elucidate the possible interrelationships between wellbeing with weight and glycemic control.

There are some limitations to this study. The exclusion criteria precluded those with co-morbidities and diabetes-related complications, thus limiting the ability to generalize our findings to a wider T2D population with poor diabetes control and state. The influence of the moderate intensity exercise performed by our participants on our results cannot be overlooked. A systematic review evaluating the effect of various types of exercise on the QoL in T2D concluded that moderate intensity aerobic exercise of ≥150 min/week significantly improved QoL compared to the control groups without an exercise intervention [41]. Although we lacked a non-exercising arm, both groups achieved and maintained similar levels of exercise throughout the study. Therefore, while we acknowledge that it is highly likely exercise has played a role in our results; dietary composition remains the key difference between the groups. Our participants received comprehensive and regular consultations with a dietitian throughout the study which may have exerted a positive effect on some aspects of psychological wellbeing. However this service is not readily available in the community and therefore may also limit the ability to generalize our findings to a wider community based T2D population. As the sample size and study duration was based on the primary outcome (HbA1c), it is acknowledged that this present study may have lacked power to detect changes for some psychological wellbeing outcomes between the intervention groups. Future larger studies of a longer duration are needed. Also, information regarding menopausal status for the female participants was not collected in this study and is a limitation. As perimenopausal and postmenopausal women have an increased risk of greater symptoms of depression and anxiety respectively [42], it is not possible to know if this has had an effect on our results.

Strengths of this study include the comprehensive use of both generic and disease-specific questionnaires. The benefits for incorporating both types of questionnaires to assess health related QoL in diabetes has been demonstrated when comparing the SF-36 and D-39 questionnaires and finding they captured different paradigms of QoL thus complementing rather than substituting for each other [43]. A high degree of weight stabilization was achieved during the weight maintenance period while maintaining exercise and allocated diet composition which afforded two distinctive phases to assess outcomes, and the opportunity to evaluate diet effects independent of weight loss.

## Conclusions

In overweight and obese adults with T2D, considerable improvements in several psychological wellbeing and HRQoL outcomes were seen in response to a modest weight loss and substantial improvements in HbA1c. These were achieved on both isocaloric HP and HC diets combined with exercise. Achieving better glycemic control appears to play an important role in improving psychological wellbeing outcomes. A HP diet may be more beneficial in promoting greater improvements in feelings of vitality during weight loss. Furthermore, when weight loss was sustained for 12 weeks, many of these benefits were maintained. This is likely to be of clinical relevance. With the growing focus on addressing HRQoL in T2D, it is imperative that specific lifestyle changes which support the physiological and psychological aspects of T2D management are implemented. Further studies that comprehensively examine the long-term effects of a variety of dietary approaches on mental wellbeing in individuals with T2D are warranted.

## Abbreviations

BGL: Blood glucose level
BMI: Body mass index
D-39: Diabetes quality of life questionnaire-39
Esn: Energy
HbA1c: Glycosylated haemoglobin
HC: Higher-carbohydrate, lower-protein diet
HP: Higher-protein, lower-carbohydrate diet
HRQoL: Health-related quality of life
sKg: Kilogram
LSEQ: Leeds sleep evaluation questionnaire
MCS: Mental component summary
PAID: Problem areas in diabetes questionnaire
PCS: Physical component summary
PSS-10: Perceived stress scale-10
QoL: Quality of life
QOS: Quality of sleep
SF-36: Short form-36
T2D: Type 2 diabetes
